# Reduced susceptibility in leptospiral strains of bovine origin might impair antibiotic therapy

**DOI:** 10.1017/S0950268818002510

**Published:** 2018-09-04

**Authors:** L. Correia, A. P. Loureiro, W. Lilenbaum

**Affiliations:** Departamento de Microbiologia e Parasitologia, Laboratório de Bacteriologia Veterinária, Universidade Federal Fluminense, Niterói, Rio de Janeiro, 24210-130, Brazil

**Keywords:** Antimicrobial, cattle, *Leptospira*, reduced susceptibility, Sejroe

## Abstract

Leptospirosis is a worldwide zoonotic disease determined by pathogenic spirochetes of the genus *Leptospira*. The control of bovine leptospirosis involves several measures including antibiotic treatment of carriers. Despite its importance, few studies regarding antimicrobial susceptibility of strains from bovine origin have been conducted. The aim of this study was to determine the *in vitro* susceptibility of *Leptospira* strains obtained from cattle in Rio de Janeiro, Brazil, against the main antibiotics used in bovine veterinary practice. A total of 23 *Leptospira* spp. strains were investigated for minimum inhibitory concentrations (MICs) and minimum bactericidal concentrations (MBCs) using broth macrodilution. At the species level, there were not differences in MIC susceptibility except for tetracycline (*P* < 0.05). Nevertheless, at the serogroup level, differences in MIC were observed among Sejroe strains, mainly for ceftiofur, doxycycline and in MBC for streptomycin (*P* < 0.05). One strain presented MBC values above maximum plasmatic concentration described for streptomycin and was classified as presenting reduced susceptibility. Efficacy of antimicrobial therapy on bovine leptospirosis could be compromised due to occurrence of infection by *Leptospira* strains presenting reduced susceptibility.

## Introduction

Leptospirosis is a worldwide zoonotic disease determined by pathogenic spirochetes of the genus *Leptospira*. Currently, *Leptospira* are classified genetically into 23 species such as pathogenic (10), saprophytes (7) and intermediates (6) species [1, 2]. Moreover, species are serologically classified into serogroups (sg) and serovars (sv), useful for serodiagnosis and for epidemiological understanding at a regional or population level [3].

In cattle, this disease is characterised mainly by reproductive problems such as infertility, prolonged intervals between births, abortion and occurrence of stillbirths, leading to important economic losses [4, 5]. Recently, this agent has been identified in the uterus of non-pregnant cows, and infection of the reproductive tract may be the most important manifestation in pathogenesis of bovine leptospirosis [6]. Leptospires from sg Sejroe are the major agents of bovine leptospirosis, and members of that sg are distributed among different species and genotypes, such as *Leptospira interrogans* (strain Hardjoprajitno), *L. borgpetersenii* (strain Hardjobovis) and *L. santarosai* (strain Guaricura) [7].

Regarding control of bovine leptospirosis, this is based on vaccination, environmental changes and antibiotic therapy [8–10]. Vaccines (bacterins) confer a limited immune response and are inefficient to prevent renal carrier status [11–13]. Antibiotic therapy is referred to as a more effective way to reduce the risk of transmission of leptospirosis in herds when associated with vaccination [9]. It is often used at the beginning of the programme to reduce the number of infected animals and to minimise the urinary shedding and consequent cow-to-cow transmission [4], as well as during the quarantine period [9].

Streptomycin is largely used for removing renal carrier status, and treatment should also be considered when reproductive failure occurs, or during an outbreak [4, 14]. Furthermore, for acute infection a combination of penicillin and streptomycin is referred to as the treatment of choice. Other antimicrobials such as ampicillin, amoxicillin, tetracycline or third-generation cephalosporin (ceftiofur) are also used with satisfactory results [4]. Oxytetracycline is reported as effective for resolving leptospirosis in countries where streptomycin was banned to livestock, like USA and Australia [14, 15].

Currently, the correlation between clinical breakpoints and clinical outcomes of antimicrobial agents is not clearly established in leptospirosis [16]. For the pharmacokinetics and pharmacodynamic ratio (PK/PD), it is necessary to understand the mechanisms of action of each antimicrobial drug and its interactions with the host, which will lead to its antimicrobial activity [17, 18]. According to Becker [19], the efficiency of an antimicrobial drug in a given infection should be based on three variables: (i) the minimum inhibitory concentration (MIC) to 90% of the pathogen, as the concentration capable of destroying 90% of the total of a microorganism (MIC_90_); (ii) its maximum plasma level (*C*_max_) and (iii) its elimination half-life. It should be emphasised that these variables are affected by the dose as well as the route of administration of the drug and the site of infection [17, 20].

Few studies focused on antimicrobial susceptibilities of *Leptospira* strains, probably due to difficulty in culturing and the slow-growing of *Leptospira* from biological samples [16]. *L. interrogans* from human origin is the most studied species and slight variations in susceptibility of strains have been reported [14, 21–23]. Nevertheless, knowledge about resistance of strains of animal origin, particularly cattle, is very limited [16, 24]. In the last few years, our group has been successful in obtaining isolates from animal origin in Brazil, particularly bovines [25]. In this context, the aim of this study was to determine the *in vitro* susceptibility of *Leptospira* strains obtained from cattle in Rio de Janeiro, Brazil against the main antibiotics used in bovine veterinary practice.

## Methods

### *Leptospira* strains and culture conditions

Leptospiral strains were originally obtained from clinical samples (urine and vaginal fluid) of cattle on several projects developed by our group from 2012 to 2016 (Table 1). Currently, they belong to the Collection of Cultures of Leptospires of Animal Origin (http://www.labv.uff.br) and are maintained in liquid nitrogen. Strains (*n* = 23) were thawed and maintained in Ellinghausen–McCullough–Johnson–Harris medium (EMJH – BD Difco™, Franklin Lakes, NJ, USA) at 30 °C and were all free of contamination or autoagglutination.

**Table 1.** Leptospiral strains (collection of cultures of leptospires of animal origin of LABV) of bovine origin tested in this study

No.	Strain	Species	sg	Clinical sample	Reference
1	U152/2013	*L. santarosai*	Shermani	Urine	[26]
2	U278/2013	*L. santarosai*	Shermani	Urine	[26]
3	2014_U76	*L. santarosai*	Sejroe	Urine	[7]
4	2014_VF237	*L. santarosai*	Sejroe	Vaginal fluid	[27]
5	2015_U237	*L. santarosai*	Sejroe	Urine	[27]
6	2014_VF66	*L. santarosai*	Sejroe	Vaginal fluid	[7]
7	2013_VF52	*L. santarosai*	Sejroe	Vaginal fluid	[7]
8	2014_U140	*L. santarosai*	Sejroe	Urine	[7]
9	U214/2013	*L. santarosai*	Sejroe	Urine	[26]
10	2014_U81	*L. santarosai*	Sejroe	Urine	[7]
11	2015_U222	*L. santarosai*	Undefined	Urine	[27]
12	2014_U83	*L. santarosai*	Tarassovi	Urine	[25]
13	U233/2013	*L. santarosai*	Grippotyphosa	Urine	[26]
14	U280/2013	*L. santarosai*	Grippotyphosa	Urine	[26]
15	U160/2013	*L. santarosai*	Sarmin	Urine	[26]
16	2014_U213	*L. santarosai*	Undefined	Urine	[27]
17	2014_U93	*L. noguchii*	Australis	Urine	Unpublished
18	2014_U65	*L. noguchii*	Australis	Urine	[27]
19	U232/2013	*L. noguchii*	Autumnalis	Urine	[28]
20	2014_U79	*L. noguchii*	Autumnalis	Urine	Unpublished
21	U73/2013	*L. noguchii*	Panama	Urine	[28]
22	2015_U349	*L. interrogans*	Sejroe	Urine	[25]
23	2015_U376	*L. interrogans*	Sejroe	Urine	[25]

### Antimicrobial agents

Stock antimicrobial solutions of 1 mg/l (or 1000 units of penicillin G/ml) of penicillin G, tetracycline, doxycycline, ceftiofur and streptomycin (Sigma-Aldrich, St. Louis, MO, USA) were prepared with specific diluents as recommended by Clinical and Laboratory Standards Institute [29]. Stock antimicrobial solutions were stored at −20 °C and divided into one-time-use aliquots.

### Macrodilution

Broth macrodilution for MICs and minimum bactericidal concentrations (MBC) was performed as recommended [21, 30]. Positive and negative controls (culture of each strain without antibiotics and EMJH without adding leptospires or antibiotics, respectively) were set up for each batch. The test was repeated twice for each strain. Serial twofold dilutions of antibiotics in EMJH medium resulted in final concentrations of 100–0.01 mg/l (for penicillin G: units/l). *Leptospira* concentration in the inoculum was determined using a Petroff-Hausser counting chamber and dark-field microscopy. Inoculum was added in order to produce a final concentration of 10^6^ leptospires/ml (final volume 2 ml). Culture tubes were incubated at 30 °C for 7 days and then examined for the presence or absence of visible growth as evidenced by turbidity and dark-field microscopy. For this, a drop of each culture tube was examined by dark-field microscopy in order to ensure the presence or absence of leptospires and their actively motile or not. Lowest concentration without growth was recorded as the MIC. After MIC determination, 10 µl was transferred from tubes without visible growth into 2 ml of EMJH medium and incubated for 3 weeks at 30 °C. The lowest antibiotic concentration that provided no visible growth as evidenced by turbidity and dark-field microscopy evaluation after 3 weeks was documented as the MBC.

### Statistical analysis

Results obtained from MIC and MBC of each strain were annotated and compared at the level of genus, species and sg. In addition, strains of the Sejroe sg were analysed between themselves. In addition, the genus was compared with MIC_90_ and MBC_90_. At the genus level, the frequency distribution of MIC and MBC values was evaluated, following the model proposed by EUCAST [31]. MIC and MBC values were analysed using the weighted Spearman correlation method with Monte Carlo simulation [32] for species, sgs and strains (sg Sejroe). Data were analysed using SPSS 22 Statistics Base software (IBM) and significance was set at *P* < 0.05. To estimate a possible *in vivo* susceptibility profile for each antimicrobial drug, the MBC values were compared with the *C*_max_ and evaluated by frequency distribution. Characteristics of the main antibiotics used in veterinary practice in bovines are presented in the Supplementary Table S1.

## Results

There were no differences in MIC susceptibility at the species level except for tetracycline (*P* < 0.05). At the genus level, antimicrobial drugs with bactericidal activity (penicillin G, ceftiofur and streptomycin) presented the lowest dispersion of the values as well as the smaller medians, in contrast to bacteriostatic antimicrobial drugs (doxycycline and tetracycline). No variation in duplicates of each strain was observed and the frequency distribution of MIC and MBC values, following the model proposed by EUCAST [31], is presented in Table 2.

**Table 2.** Distribution and median of MIC and MBC for five antimicrobial agents among the 23 *Leptospira* sp. from bovine origin

Antimicrobial agent	No. of strains with MIC (mg/l) of:															Median
	0.01	0.02	0.05	0.1	0.2	0.39	0.78	1.56	3.13	6.25	12.5	25	50	100	>100	
Penicillin G	5	7	2	5		3	1									0.02
Ceftiofur				1	4	9	2	4			3					0.39
Streptomycin						5	6	5	5		1		1			1.56
Doxycycline					2	4	2	8	4	2	1					1.56
Tetracycline			1		2	6		4	4	5	1					1.56
	No. of strains with MBC (mg/l) of:															
	0.01	0.02	0.05	0.1	0.2	0.39	0.78	1.56	3.13	6.25	12.5	25	50	100	>100	
Penicillin G				9		1	2	2	1	4	1	2			1	0.78
Ceftiofur						2	1	5	4	2	5	1	2		1	3.13
Streptomycin						1	2	5	5	3	3	2	1		1	3.13
Doxycycline						1			1	5	7	5	3	1		12.5
Tetracycline								1	1	3	7	8	2		1	12.5

At the sg level there was no significant difference. However, strains of leptospires belonging to the same sg (Sejroe) from different species, *L. interrogans* (two strains) and *L. santarosai* (eight strains) showed differences in their antimicrobial susceptibility, being these differences observed in the MIC values for ceftiofur (*P* = 0.025) and doxycycline (*P* = 0.032) and in MBC values of streptomycin (*P* = 0.022). When MIC_90_ and MBC_90_ values of antimicrobial agents were compared, penicillin G and streptomycin were the drugs with the lowest values. MIC and MBC values for each strain, including MIC_90_ and MBC_90_ values for the genus (MICs and MBCs which at least 90% of strains tested in a specific group are inhibited) are shown in Table 3.

**Table 3.** Susceptibility results (MICs and MBCs in mg/l) of antibiotics tested for strains of *Leptospira* sp. from bovine origin

MIC (MBC)								
No.	Strain	Species	sg	Penicillin	Ceftiofur	Streptomycin	Doxycycline	Tetracycline
1	U152/2013	*L. santarosai*	Shermani	0.1 (0.1)	1.56 (3.13)	3.13 (25)	6.25 (25)	6.25 (12.5)
2	U278/2013	*L. santarosai*	Shermani	0.01 (0.1)	12.5 (12.5)	50 (50)	1.56 (100)	12.5 (12.5)
3	2014_U76	*L. santarosai*	Sejroe	0.78 (25)	0.39 (50)	0.78 (12.5)	6.25 (6.25)	6.25 (6.25)
4	2014_VF237	*L. santarosai*	Sejroe	0.39 (25)	1.56 (25)	3.13 (6.25)	3.13 (12.5)	1.56 (12.5)
5	2015_U237	*L. santarosai*	Sejroe	0.1 (0.39)	0.39 (1.56)	0.39 (1.56)	1.56 (6.25)	1.56 (6.25)
6	2014_VF66	*L. santarosai*	Sejroe	0.02 (6.25)	0.39 (6.25)	1.56 (12.5)	1.56 (6.25)	3.13 (>100)
7	2013_VF52	*L. santarosai*	Sejroe	0.02 (0.78)	0.78 (3.13)	1.56 (1.56)	1.56 (25)	3.13 (25)
8	2014_U140	*L. santarosai*	Sejroe	0.01 (1.56)	0.39 (50)	1.56 (3.13)	0.39 (50)	0.39 (25)
9	2013_U214	*L. santarosai*	Sejroe	0.1 (12.5)	0.39 (>100)	3.13 (6.25)	1.56 (12.5)	1.56 (25)
10	2014_U81	*L. santarosai*	Sejroe	0.05 (0.78)	0.2 (0.78)	0.39 (1.56)	0.78 (3.13)	0.39 (3.13)
11	2015_U222	*L. santarosai*	undefined	0.02 (0.1)	0.39 (0.39)	3.13 (3.13)	3.13 (12.5)	6.25 (25)
12	2014_U83	*L. santarosai*	Tarassovi	0.01 (0.1)	0.39 (0.39)	1.56 (1.56)	0.39 (0.39)	0.39 (1.56)
13	U233/2013	*L. santarosai*	Grippotyphosa	0.02 (0.1)	1.56 (1.56)	0.78 (0.78)	3.13 (6.25)	3.13 (12.5)
14	U280/2013	*L. santarosai*	Grippotyphosa	0.39 (6.25)	12.5 (12.5)	1.56 (3.13)	1.56 (50)	6.25 (50)
15	U160/2013	*L. santarosai*	Sarmin	0.39 (6.25)	0.78 (1.56)	0.78 (3.13)	0.39 (12.5)	0.05 (6.25)
16	2014_U213	*L. santarosai*	undefined	0.1 (0.1)	0.39 (1.56)	0.78 (0.78)	3.13 (25)	6.25 (25)
***L. santarosai* range**				**0.01–0.78 (0.1–25)**	**0.2–12.5 (0.39–>100)**	**0.39–50 (0.78–50)**	**0.39–6.25 (0.39–100)**	**0.05–12.5 (1.56–>100)**
17	2014_U93	*L. noguchii*	Australis	0.05 (0.1)	1.56 (6.25)	3.13 (6.25)	1.56 (12.5)	3.13 (12.5)
18	2014_U65	*L. noguchii*	Australis	0.01 (0.1)	12.5 (12.5)	12.5 (12.5)	12.5 (12.5)	1.56 (25)
19	U232/2013	*L. noguchii*	Autumnalis	0.02 (3.13)	0.2 (1.56)	0.78 (1.56)	0.78 (12.5)	0.39 (12.5)
20	2014_U79	*L. noguchii*	Autumnalis	0.1 (0.1)	0.39 (3.13)	0.39 (0.39)	1.56 (6.25)	0.39 (50)
21	U73/2013	*L. noguchii*	Panama	0.1 (6.25)	0.2 (3.13)	0.78 (3.13)	0.2 (25)	0.2 (12.5)
***L. noguchii* range**				**0.01–0.1 (0.1–6.25)**	**0.2–12.5 (1.56–12.5)**	**0.39–12.5 (0.39–12.5)**	**0.2–12.5 (6.25–25)**	**0.2–3.13 (12.5–50)**
22	2015_U349	*L. interrogans*	Sejroe	0.02 (>100)	0.2 (12.5)	0.39 (>100)	0.2 (25)	0.2 (25)
23	2015_U376	*L. interrogans*	Sejroe	0.02 (1.56)	0.1 (12.5)	0.39 (25)	0.39 (50)	0.39 (25)
***L. interrogans* range**				**0.02 (1.56–>100)**	**0.1–0.2 (12.5)**	**0.39 (25–>100)**	**0.2–0.39 (25–50)**	**0.2–0.39 (25)**
**MIC_90_ (MBC_90_) *Leptospira* sp.**				**0.39 (25)**	**12.5 (50)**	**3.13 (25)**	**6.25 (50)**	**6.25 (50)**

When comparing the *C*_max_/MBC values for each antimicrobial drug evaluated, it was observed that for some studied drugs the *C*_max_ was insufficient for achieving the MBC values of some strains, and in those cases, strains were defined as presenting reduced susceptibility. Of the studied strains, only one showed susceptibility to all antimicrobials studied (2014_U83 strain). One strain had reduced susceptibility to one antimicrobial, two had reduced susceptibility to two antimicrobials, 13 strains presented reduced susceptibility to three antimicrobials, five showed reduced susceptibility to four antimicrobials and one strain to all antimicrobials tested (2015_U349 strain). Reduced susceptibility was observed in 22/23 for doxycycline (95.6%), 20/23 for ceftiofur (86.9%), 18/23 for tetracycline (78.2%), 8/23 for penicillin G (34.8%) and one strain (4.35%) for streptomycin, *L. interrogans* sg Sejroe (2015_U349 strain).

## Discussion

Differences in susceptibility have already been reported among *Leptospira* species of human origin [21–23, 33, 34], as well as strains from dogs and rats [30] and from various animals [16, 24, 35]. To our knowledge there is no study focusing exclusively on strains from bovine origin, which impairs the comparison of our results with other regions. Recently, a study reported heterogeneity of MIC for leptospiral strains from food-producing animals and the need of additional studies to better discriminate between susceptible and resistant *Leptospira* strains of animal origin was raised [16].

In the current study, in general, MIC and MBC values of penicillin G were lower compared with other antimicrobials, which agree with other studies [14, 16, 24, 30]. Similarly, the MIC results for ceftiofur and streptomycin observed in this study are similar to those already described in the literature [16, 24]. Noteworthy that MBC values as high as >100 units/l for penicillin and >100 mg/l for streptomycin were observed for two strains, one *L. santarosai* and one *L. interrogans*, which has already been reported for *L. interrogans* strains of human origin [33].

Considering doxycycline and tetracycline, high MICs were necessary for growth inhibition, which corroborates with other studies conducted with the strains of animal origin [16, 24]. Since they are bacteriostatic antimicrobials, their use should be cautious, considering that they are not the best option for treatment. In addition, tetracyclines are excessively used in in animal production as growth promoters and to improve food efficiency, which may have contributed to the emergence of antimicrobial resistance and low susceptibility to these agents [16]. Moreover, the presence of gene related to tetracycline resistance (*tet*A) has already been demonstrated in leptospiral DNA [36].

An important result of the current study was that strains belonging to sg Sejroe present significant differences in antimicrobial susceptibility against streptomycin. Members of this sg, such as *L. borgpetersenii* sv Hardjo (Hardjobovis) and *L. interrogans* sv Hardjo (Hardjoprajitno) are worldwide known as major agents of bovine leptospirosis [4]. Recently, strains of *L. santarosai* sv Guaricura have been also reported to be disseminated among cattle in South America [7], which reinforces the need of antimicrobial susceptibility evaluation of those strains in order to improve the control of leptospirosis.

Nevertheless, *in vitro* results not always have clinical significance, which is only understood when compared with the PK and PD characteristics of each antimicrobial drug [19]. This is particularly difficult to do on leptospires, since definition of breakpoints is still missing [16, 31]. It has been suggested for other bacteria that the MBC values of each drug when confronted with plasma concentration (*C*_max_) may be useful for predicting the effectiveness of antibiotics [17].

In this context, regarding the possibility of therapeutic failure, reduced susceptibility was observed for ceftiofur in three leptospiral species. Therefore, infection in cattle may require higher antimicrobial doses than is generally recommended in infection by that strains. Indeed, those findings may explain the results observed *in vivo* by Alt and collaborators [14], who proposed that antibiotic therapy with ceftiofur should be performed with higher dosage than the usually recommended (20 mg/kg, IM, once daily for 3 days or 5 mg/kg, IM, once daily for 5 days).

Moreover, one-third of the strains presented reduced susceptibility to penicillin G, which makes that drug a poor choice for the treatment of bovine leptospirosis. Although it is rarely used as a single drug for the treatment of bovine leptospirosis, it is commonly combined with streptomycin, and satisfactory results have been reported [4, 14]. For tetracyclines, a high rate of strains with reduced susceptibility is observed. It has been stated that a single dose of oxytetracycline (20 mg/kg, IM) can eliminate kidney carrier status [14] and in outbreaks an integrated approach based on enhanced biosafety measures, herd-level antimicrobial treatment using oxytetracycline and vaccination was effective [9].

Finally, the main point of this study refers to the reduced susceptibility to streptomycin in leptospiral strains of bovine origin. A single dose (25 mg/kg) of this drug is recommended in order to eliminate the renal carrier status in cattle [4, 14, 37]. Nevertheless, results of that therapy are often frustrating, particularly in infection by strains of sg Sejroe, and persistent infection may occur even after two streptomycin doses with 15 days interval [14, 38]. In this context, the current study demonstrates that the current recommended protocol could not be adequate for all leptospiral strains. Thus, eventual failure in treatment may be due to the reduced susceptibility (MBC value of the strain higher than drug plasmatic concentration) of some infecting strains. Although resistance is not frequently reported in leptospires, the occurrence of streptomycin-resistant mutants *in vitro* in saprophytic and pathogenic species has been described and is related to a mutation in the *rps*L gene [39]. Therefore, the demonstration of leptospiral strains of bovine origin with reduced susceptibility to streptomycin must be taken into account in order to avoid, as happened in other infectious agents, the selection of resistant mutants that may become important emerging pathogens in the future.

Since leptospires can colonise different tissues and organs, MBC was primarily compared with plasmatic concentrations of the drugs. Nevertheless, although other sites of infection have been described, such as uterus and vaginal tract [6], renal tubules are still referred to as the main site of colonisation of infected cattle. In this context, when compared with kidney and urinary concentrations of the drugs, all strains are susceptible to penicillin, but strains with reduced susceptibility to streptomycin and tetracycline still occur (Supplementary Table S2).

In conclusion, our results demonstrate that streptomycin can still be considered as the best choice of antimicrobial agent for treatment of bovine leptospirosis. Nevertheless, therapy on bovine leptospirosis could be impaired due to the occurrence of infection by *Leptospira* strains presenting reduced susceptibility to that drug.

## Supplementary material

For supplementary material accompanying this paper visit https://doi.org/10.1017/S0950268818002510. click here to view supplementary material
