# Supplementary material for: Reduced susceptibility in leptospiral strains of bovine origin might impair antibiotic therapy
Source: Epidemiol Infect. 2018 Sep 4;147:e5. doi: 10.1017/S0950268818002510 (PMC6518573; doi:10.1017/S0950268818002510)
Supplement: Supplementary file 1 [file S0950268818002510sup001.doc]

Epidemiology and Infection

**Reduced susceptibility in leptospiral strains of bovine origin might impair antibiotic therapy**

L. CORREIA, A. P. LOUREIRO, W. LILENBAUM*

**Supplementary Table**

**Supplementary Table 1.** Characteristics and reference values for cattle of each antimicrobial tested.

| **Antimicrobial Agent** | **Antimicrobial Activity** | **Commercial dosage** | ***Cmax*** | **Dosage in literature** | **Route of Administration** | **Reference** |
| --- | --- | --- | --- | --- | --- | --- |
| Penicillin G | Bactericide | 25000 UI/kg | 3.42 UI/L | 28000 UI/kg | Intramuscular | [1] |
| Ceftiofur | Bactericide | 1 mg/kg | 1.0 mg/L | 3 mg/kg | Intravenous | [2] |
| Streptomycin | Bactericide | 25 mg/kg | 78 mg/L | 25 mg/kg | Intramuscular | [3] |
| Doxycycline | Bacteriostatic | 4 mg/kg | 1.99 mg/L | 10 mg/kg | Intravenous or Intramuscular | [4] |
| Tetracycline | Bacteriostatic | 5 mg/kg | 6.8 mg/L | 10 mg/kg | Intramuscular | [5] |

**Supplementary Table 2.** Concentration values in kidney and urine for cattle of antimicrobial tested.

| **Antimicrobial Agent** | **Kidney concentration** | **Urine concentration** | **Reference** |
| --- | --- | --- | --- |
| Penicillin G | 16.7 mg/kg | 351 UI/mL | [1,6] |
| Ceftiofur | 5.54 mg/kg | ND | [7] |
| Streptomycin | 6.61 mg/kg | 1.7 mg/L | [8] |
| Doxycycline | ND | ND | ND |
| Tetracycline | 1 mg/kg | 0.90 mg/L | [6] |

ND: no data achieved

**Supplementary References**

1. **Dubreuil P, *et al.*** Penicillin concentrations in serum, milk, and urine following intramuscular and subcutaneous administration of increasing doses of procaine penicillin G in lactating dairy cows. *Canadian Journal of Veterinary Research* 2001; **65**: 173–180.

2. **Erskine RJ, *et al.*** Ceftiofur distribution in serum and milk from clinically normal cows and cows with experimental *Escherichia coli*-induced mastitis. *American Journal of Veterinary Research* 1995; **56**: 481–485.

3. **Stalheim O**. Absorption and Excretion of Tritiated Dihydrostreptomycin in Cattle and Swine. *Am J Vet Res.* 1970; **31**: 497–500.

4. **Vargas-Estrada D, Gracia-Mora J, Sumano H**. Pharmacokinetic study of an injectable long-acting parenteral formulation of doxycycline hyclate in calves. *Research in Veterinary Science* 2008; **84**: 477–482.

5. **Mevius DJ, *et al.*** Comparative pharmacokinetics, bioavailability and renal clearance of five parenteral oxytetracycline-20% formulations in dairy cows. *The Veterinary quarterly* 1986; **8**: 285–294.

6. **Food and Agriculture Organization of the United Nations**. Residues of some veterinary drugs in animals and foods. Thirty-sixth Meeting of the Joint FAO/WHO Expert Committee on Food Additives. *FAO Food Nutr Pap.* 1991; **41(3)**: 1-119.

7. **Food and Agriculture Organization of the United Nations**. Residues of some veterinary drugs in animals and foods. Joint FAO/WHO Expert Committee on Food Additives. *FAO Food Nutr Pap.* 1996; **41(8)**: 1-166.

8. **Food and Agriculture Organization of the United Nations**. Residues of some veterinary drugs in animals and foods. Forty-third meeting of the Joint FAO/WHO Expert Committee on Food Additives. *FAO Food Nutr Pap*. 1995; **41(7)**: 1-112.
